# Antioxidant Potential of Aqueous Dispersions of Fullerenes C_60_, C_70_, and Gd@C_82_

**DOI:** 10.3390/ijms22115838

**Published:** 2021-05-29

**Authors:** Ivan V. Mikheev, Madina M. Sozarukova, Dmitry Yu. Izmailov, Ivan E. Kareev, Elena V. Proskurnina, Mikhail A. Proskurnin

**Affiliations:** 1 Analytical Chemistry Division, Chemistry Department, Lomonosov Moscow State University, 119991 Moscow, Russia; s_madinam@bk.ru (M.M.S.); proskurnin@gmail.com (M.A.P.); 2 Kurnakov Institute of General and Inorganic Chemistry, Russian Academy of Sciences, 119991 Moscow, Russia; s_madinam@bk.ru; 3 Faculty of Fundamental Medicine, Lomonosov Moscow State University, 119234 Moscow, Russia; dizm@mail.ru; 4 Institute of Problems of Chemical Physics of the Russian Academy of Sciences, 142432 Moscow, Russia; kareev@icp.ac.ru; 5 Research Centre for Medical Genetics, 115522 Moscow, Russia; proskurnina@gmail.com

**Keywords:** fullerene, endofullerene, aqueous fullerene dispersion, antioxidant capacity, antioxidant activity, chemiluminometry

## Abstract

The antioxidant potential (capacity and activity) of aqueous fullerene dispersions (AFD) of non-functionalized C_60_, C_70_, and Gd@C_82_ endofullerene (in micromolar concentration range) was estimated based on chemiluminescence measurements of the model of luminol and generation of organic radicals by 2,2′-azobis(2-amidinopropane) dihydrochloride (ABAP). The antioxidant capacity was estimated by the TRAP method, from the concentration of half-suppression, and from the suppression area in the initial period. All three approaches agree and show that the antioxidant capacity of AFDs increased in the order Gd@C_82_ < C_70_ < C_60_. Mathematical modeling of the long-term kinetics data was used for antioxidant activity estimation. The effect of C_60_ and C_70_ is found to be quenching of the excited product of luminol with ABAP-generated radical and not an actual antioxidant effect; quenching constants differ insignificantly. Apart from quenching with a similar constant, the AFD of Gd@C_82_ exhibits actual antioxidant action. The antioxidant activity in Gd@C_82_ is 300-fold higher than quenching constants.

## 1. Introduction

Water-soluble fullerene species are promising for various medical applications, and they have been proposed as vital components for humans and environmental systems [1]. Fullerenes and, in particular, their water-soluble derivatives, are considered radical scavenging agents [2], possess antioxidant activity [3], acquire remarkable antimicrobial properties [4], cytotoxicity [5], DNA cleavage, and lipid peroxidation mediated by reactive oxygen species (ROS) [6].

The investigation of non-functionalized (without addends) aqueous fullerene dispersions (AFD), which are produced by ultrasound-assisted solvent exchange [7], dialysis [8], or direct ultrasonic treatment [9], is developing widely. The mechanism of AFD stabilization is not fully understood, but attempts have been made to explain it by hydroxylation of the fullerene cage [10]. Recently, substantial advances have been made in areas of colloidal fullerene properties [11], physicochemical interactions at the nano–bio interface [12], biological mechanisms and physicochemical characteristics responsible for driving fullerene toxicity [13], and biological activity of water-soluble fullerene adducts [14].

The recent review discussed [15] that fullerenes possess an inert scaffold with antioxidant functionalities. C_60_ is a very weak chain-breaking antioxidant with an inherent rate constant for trapping peroxyl radicals per se (*k*_inh_=0.3 × 10^3^ M^−1^ s^−1^). However, some antioxidants covalently bound to fullerenes increase antioxidant activity insignificantly. Grafting their cage with small-molecule antioxidant moieties such as synthetic phenols (2,6-di-tert-butyl-4-methylphenol) broadens their antioxidant potential conveying peroxyl radical-trapping activity up to 30 times [16]. C_60_ conjugated with phenols indicates a significant improvement of oxidative stability [17]. A C_60_ derivative with covalently bonded analog of α-tocopherol with hydroxychromanyl moiety is an effective antioxidant acting in model lipid matrices: saturated stearic acid and unsaturated linolenic acid during the non-isothermal oxidation tested by differential scanning calorimetry [18].

Derivatives C_60_(C(COOH)_2_)_2_, C_60_(OH)_22_, and Gd@C_82_(OH)_22_ can stabilize the mitochondrial membrane potential and reduce intracellular ROS production in the order: Gd@C_82_(OH)_22_ ≥ C_60_(OH)_22_ > C_60_(C(COOH)_2_)_2_. These derivatives scavenge the stable 2,2-diphenyl-1-picryhydrazyl radical, ROS, and inhibit lipid peroxidation in vitro [19]. The common fullerene derivatives in the structure are fullerenols, with up to 42 hydroxyl groups, depending on the fullerene type. Hydroxylation is one of the cheapest and most straightforward approaches to dissolving fullerenes in water and does not require deep purification of the resulting product. However, even minor surface derivatization may increase the antioxidant activity of fullerenes [20]. Less toxicity and greater antioxidant capacity are proven for fullerenols C_60_O_y_(OH)_x_, C_60,70_O_y_(OH)_x_, x + y = 24 ÷ 28 [21]. There are two limitations of any derivatization of fullerene cage. First, these groups can be involved in metabolic processes; they can reduce the π-electron system availability leading to reversible free radical capture [22], differently affecting the spin environment [23]. Second, fullerene derivatives could act as potent oxidizing agents under excitation with light in the presence of oxygen [24].

The radical reactivity of fullerenes is discussed [25]. First attempts at studying superoxide dismutase (SOD) mimic activity have yet to be made [26] for in vitro and cell models. It is known that unsaturated lipids are a target of free radicals. Their oxidation (lipid peroxidation) mechanism has been described and proven [6]. The result is the accumulation of lipid hydroperoxides as intermediate stable products. The ability of fullerenes to initiate lipid oxidation has not been widely assessed. There is a report on their ability to trap lipid peroxyl radicals and act as chain-breaking antioxidants [27]. The impact of fullerenes in in vivo and in vitro experiments for *Cyprinus carpio* brains confirmed the absence of lipid peroxidation [28]. The protective action of C_60_ most probably results from its ability to be included in the cell membrane and avoid lipid peroxidation [29].

The antioxidant and superoxide anion-radical (SAR) scavenging properties of non-functionalized AFDs have not been thoroughly studied [30]. The data on the antioxidant activity of unmodified fullerenes in their aqueous dispersions are almost absent. There is an ambiguity in the information about the ability of fullerenes to generate ROS. Several studies deal with C_60_ solutions stimulating ROS generation [31]. Another study evidenced the antioxidative properties of fullerenes [32]. Additionally, the possible antioxidant mechanism of fullerenes, in particular C_60_, deals with loading their molecules with protons to acquire a positive charge distributed over the fullerene. Such charge-loaded particles could be transferred through the inner membrane of mitochondria. In this case, the transmembrane potential is reduced [33], significantly reducing SAR production [34]. Furthermore, C_60_ is capable of penetrating an artificial lipid bilayer [35]. Fullerene soot C_60_ and C_70_ not only retards oxidation as an alkyl radical quencher but also operates as a peroxy radical scavenger [36] in the model reaction of initiated (2,2′-azobisisobutyronitrile, AIBN). For reactivity of C_60_ during oxidation of a series of hydrocarbons shows that the fullerene does not react with the RO_2_^•^ radicals indicate an extremely weak rate constant estimated from CL [37].

The conventional approach to describing the antioxidant properties of low molecular weight free-radical scavengers is based on a quantitative assessment of their ability to terminate free-radical chain reactions against a standard antioxidant compound. Evaluation of the antioxidant status of compounds, the total radical-trapping potential (TRAP) method, and total antioxidant reactivity (TAR) from luminol-enhanced chemiluminescence (CL) measurements have been previously developed [38]. This approach is based on the ability to trap radicals formed during the decomposition of thermolabile azo compounds. However, this technique does not consider the physicochemical parameters of the antioxidant. A more rigorous approach to the description of antioxidant properties considers the determination of the antioxidant concentration and the mathematical modeling [39] of the rate constant of the interaction with the radicals [40]. Different antioxidants result in different chemiluminescence curves, making it impossible to use any single parameter to characterize the activity of substances of different chemical nature [41].

The antioxidant potential is an umbrella term to quantitatively describe the thermodynamic and kinetic aspects of the antioxidant action [42]. In this work, we assessed both the thermodynamic *antioxidant capacity* (the total number of neutralized radicals per unit of fullerene concentration) by quantitative comparison with Trolox^®^ as a reference compound [38]. We also applied mathematical modeling to estimate rate constants of fullerenes, i.e., the kinetic *antioxidant activity* (the dynamic interception ability of radicals) [39]. As far as we are concerned, kinetics modeling for fullerenes were not used previously.

Thus, this paper deals with the antioxidant potential of aqueous fullerene dispersions of C_60_, C_70_, and Gd@C_82_ as both antioxidant capacity and antioxidant activity using various approaches, including computer simulation. The antioxidant potential of AFDs was estimated using luminol-enhanced chemiluminescence with 2,2’-azobis(2-amidinopropane) dihydrochloride (ABAP, 2.5 mM) as a source of free radicals at 37 °C in a phosphate buffer solution (100 mM, pH 7.4).

## 2. Results

### 2.1. Assessment of the Antioxidant Capacity of AFDs

**Figure 1:**
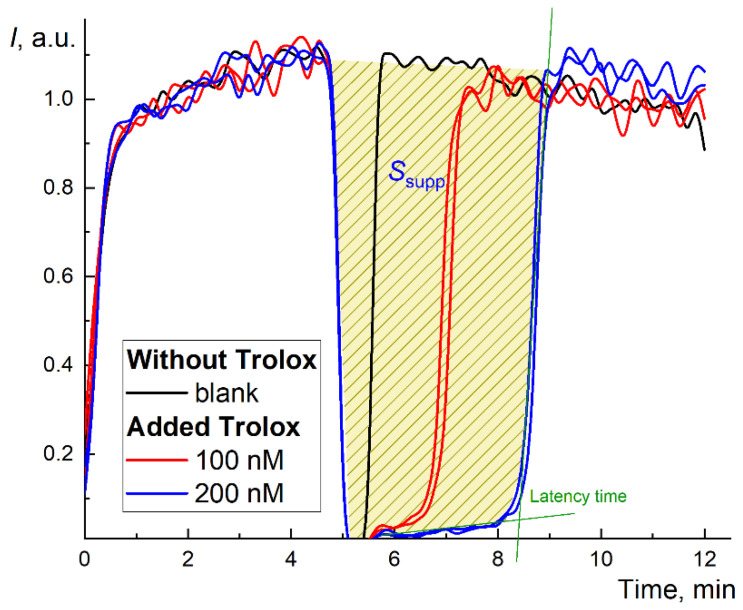
Long-term chemiluminograms of a strong antioxidant Trolox^®^ in the system 2.5 mM ABAP and 2 µM luminol up to 100 min. The figure shows the latency period and the principle of calculating the area of signal suppression (for the concentration of 200 nM, blue line).

Here, the “antioxidant potential” means both antioxidant activity and antioxidant capacity. In the technical IUPAC report [43], the terms “antioxidant capacity/activity” have not been separated, although these parameters are rather complementary. The antioxidant capacity (the number of neutralized radicals per unit of fullerene concentration) was assessed using the modified TRAP protocol [44] and TAR protocol [38]. The TRAP index is calculated from the latent period (Figure 1), while the TAR index is obtained from the rapid decrease in luminescence after adding the antioxidant. The antioxidant activity (kinetic constants of the reaction of an antioxidant with a free radical) has been determined using the computer simulation of the chemiluminescence kinetics. 

In the TRAP method, Trolox is used as a reference substance [45]. The chemiluminograms for Trolox (100 and 200 nM) are shown in Figure 1. The effect of Trolox in the ABAP/luminol system is typical for strong antioxidants: the complete suppression of chemiluminescence followed by complete depletion of the antioxidant with a rapid increase in the CL intensity to the previous stationary (blank) level after the latent period [46]. However, the latent period depends on the initial stationary CL level *I*_0_ and requires precise measurements of a short suppression period and restoration of the level *I*_0_ after the antioxidant action. Thus, it provides reliable data for strong antioxidants only.

**Figure 2:**
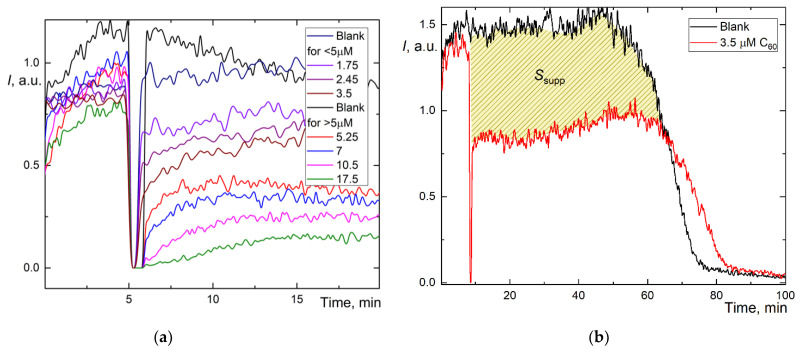

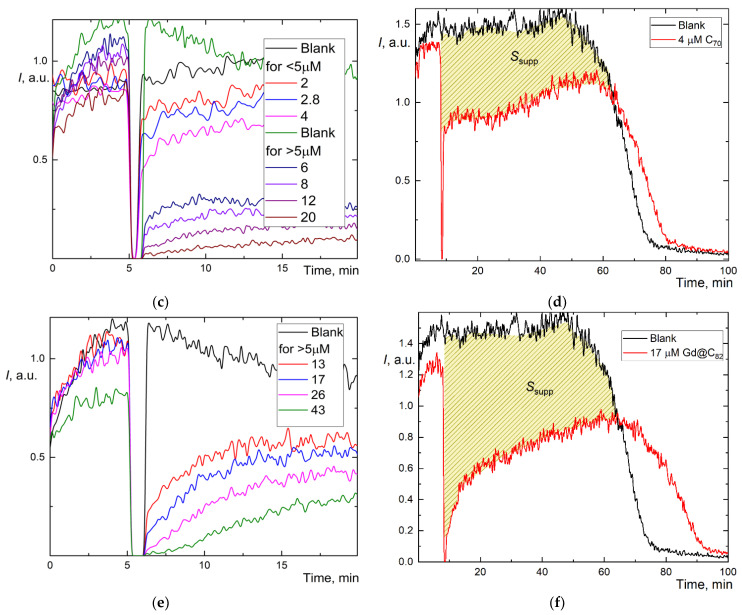
Chemiluminograms of aqueous fullerene dispersions (AFD) in 2.5 mM ABAP and 2 µM luminol (**a**) C_60_ in the concentration range of 1.8–18 μM up to 20 min; (**b**) long-term chemiluminograms of C_60_ (3.5 μM) up to 100 min; (**c**) C_70_ in a range of concentration 2.0–20 μM up to 20 min; (**d**) long-term chemiluminograms of C_70_ (4.0 μM) up to 100 min; (**e**) Gd@C_82_ in a range of concentration 4.0–40 μM; (**f**) long-term chemiluminograms of Gd@C_82_ (17 μM) up to 100 min. A sharp decrease in the signal between 5 and 10 min results from adding AFDs, the signal is not registered.

Therefore, to assess the antioxidant capacity by an alternative approach, we used the area of suppression of chemiluminescence *S* (Figure 1), which is proportional to the total number of radicals scavenged by the antioxidant, i.e., antioxidant capacity. The kinetics of the antioxidant action for fullerenes differed from Trolox (Figure 2a,c,e). Instead of almost rectangular (“trough”) suppression of the signal (Figure 1), we observed a decrease in the stationary level typical for “weak” (or relatively slow) antioxidants. Strictly speaking, it was not possible to wait until the signal returned to the stationary level for all three fullerenes (i.e., the antioxidant was consumed, Figure 2b,d,f).

However, we estimated the area of signal suppression *S*_supp_ for C_60_ and C_70_ (shaded areas in Figure 2b,d; for Gd@C_82_, we failed to calculate this area correctly as the antioxidant was not consumed during the operation of the CL model, and the CL intensity did not return to the initial level. The integration suppression area normalized to concentration (Table 1) showed the behavior, C_60_ > C_70_ > Gd@C_82_, ratios 4.3:2.4:1. The recalculation of *S*_supp_ to Trolox showed C_60_ has a 3-fold lower capacity than Trolox and Gd@C_82_, at 7% capacity compared to Trolox.

As calculating the area of suppression of chemiluminescence was unsuitable for Gd@C_82_, and with some reservations applicable for C_60_ and C_70_, we used a different method for determining the capacity. The addition of weak antioxidants leads not to the complete suppression but to a decrease in chemiluminescence intensity plateau Δ*I*. However, the dependence of Δ*I* on *c* for fullerenes proved to be nonlinear (Figure 2a,c,e), and the CL intensity does not show a stable plateau. Thus, we used an approach based on the combination of the intensity decrease and the suppression area [44]. We calculated the suppression area for the first 20 min of the reaction, *S*_20_ (Table 1). This value for low antioxidant capacities is more accurate than TRAP or TAR because the results do not depend on the initial level of chemiluminescence [44], and thus both high and low antioxidant capacities can be compared [47]. By this approach, AFDs can be ranked as C_60_ > C_70_ > Gd@C_82_ (Table 1), capacity ratios are 2.7:1.6:1.

Furthermore, for weak antioxidants, the antioxidant capacity can be estimated by the concentration of semi-suppression of the initial luminescence ($c_{1/2}$) [21]. By this approach, AFDs can be ranked as C_60_ > C_70_ > Gd@C_82_ (Table 1). Ratios of reciprocal half-suppression signal concentrations were 3.5:1.7:1.

Thus, the results of estimation by TRAP suppression area, the suppression area for the first 20 min of the reaction, *S*_20_, and $c_{1/2}$ agree with each other. These rows are also in accordance with the fraction of active molecules on the surface of fullerene clusters in AFDs, 1.8:1.6:1 [26]. As a whole, the antioxidant capacities of studied fullerenes in AFD by three approaches lie within one order of magnitude, and they show the properties of weak antioxidants.

### 2.2. Antioxidant Activity (Chemiluminescence Kinetics before Antioxidant Depletion)

A mathematical model simulating the steady-state level of chemiluminescence without antioxidants consisted of two reactions: (1) the free-radical generation from ABAP and (2) chemiluminescence reactions:

1)   ABAP → R^•^                    (constant *k*_R_), decomposition of ABAP

2)   R^•^ + Lum → RLum*      (constant *k*_Lum_), formation of the excited product

2a)  R^•^ + Lum → RLum*      luminescence

where R^•^ is a free radical or reaction product in the electronically excited state, which reacts with antioxidants, and P is the stable product of the free-radical reaction.

Fullerenes are known to be both antioxidants [48] and fluorophores [49] and act as fluorescence quenchers [50,51]. We evaluated the properties of fullerenes as quenchers for the ABAP–luminol system (Figures S1 and S2, Supplementary Materials). The Stern–Volmer constants are C_60_~C_70_ > Gd@C_82_, (3.7 ± 0.1, 3.8 ± 0.1, and 2.9 ± 0.1) × 10^4^ M^−1^, respectively, which have good accordance with the existing data [32,52,53]. The fluorescence spectra are presented in the Supplementary Materials. From these data, we expected that fullerenes might play two roles in the system: actual antioxidant action and chemiluminescence quenching. Thus, to model the action of AFDs, we took into account the following reactions:

3)   AO + R^•^ → …              (antioxidant action, constant *k*_In1_)

3a)  AO + RLum* → …      (excited product quenching, constant *k*_In2_)

Rate constants of the inhibition reactions 3 and 3a are used to prove and estimate the antioxidant activity.

**Figure 3:**
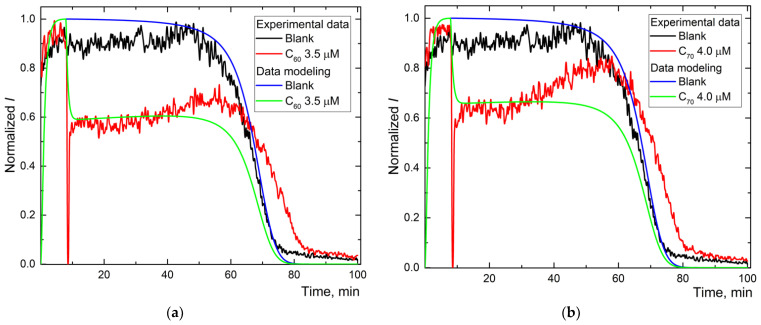

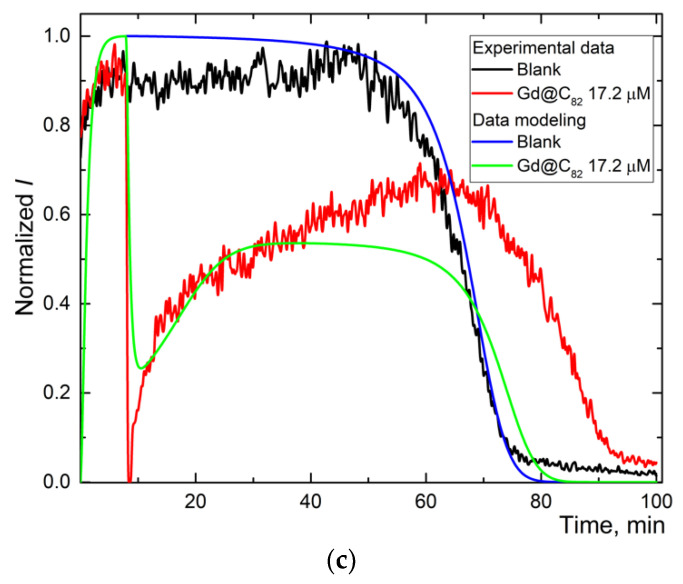
The experimental and simulated chemiluminescence plots for aqueous fullerene dispersions C_60_ (**a**), C_70_ (**b**), and Gd@C_82_ (**c**) for one-stage mechanism. Black is the blank; blue is the simulated data of the blank; red is experimental data for aqueous fullerene dispersions; and green is simulated data for aqueous fullerene dispersions.

To simulate the reaction kinetics, we recorded the chemiluminograms until complete luminol depletion [44]. All AFDs satisfy the requirements for mathematical modeling: (1) the moment of antioxidant depletion is registered, and (2) the concentration dependence is traced. To carry out the simulation, we selected the optimum concentration ranges of the investigated AFDs (close to $c_{1/2}$; Table 1). The initial simulation conditions are summed up in Table 2. The experimental and model plots for AFD are shown for C_60_ (Figure 3a), C_70_ (Figure 3b), and Gd@C_82_ (Figure 3c).

Experimental and calculated plots have a sufficient degree of coincidence. For Trolox and AFDs, recording the whole curve required ca. 100 min (Figure 2b,d,f). The simulation shows the expected values of reactions (1), (2), and (2a) and the constant of the primary antioxidant process, the radical interception reaction (3) of 10^4^ μM^−1^ min^−1^ (Table 2).

For AFDs, the rate constants of (1) and (2) differed insignificantly, while the rate constant of the luminescence process decreased by a factor of 4. Reaction (3a), the interaction of the antioxidant with the excited product of luminol was revealed for all AFDs; rates are in the order C_60_ > C_70_~Gd@C_82_, the ratio of reaction rate constants is 1.5:1:1. Along with a decrease in the rate constant of (2a), it can be considered quenching.

The reaction (3) intercepting radicals from ABAP is observed for Trolox and Gd@C_82_ AFD. The simulation shows that this process has a rate constant 300 times higher than the quenching (Table 2), while the concentration of the antioxidant calculated in the process (3) is estimated as 100 times lower than the total fullerene concentration in the AFD. The antioxidant activity of Gd@C_82_ is 300 times lower than for Trolox.

## 3. Discussion

The study [44] classified the antioxidant activity by rate constants as strong, higher than 2 μM^−1^ min^−1^, medium, higher than 0.1 μM^−1^ min^−1^, and weak, below 0.01 μM^−1^ min^−1^. Strong oxidants can be measured easily; the medium can be measured, as well. In the case of weak oxidants, calibration is complicated as the action is weak and slow and probably does not return to the pre-application stationary CL level.

C_60_, C_70_, and Gd@C_82_ are comparable and refer to medium-strength antioxidants (Table 2). On the contrary, the same constants attributed to AO + RLum* reaction for C_60_, C_70_, and Gd@C_82_ reveal that fullerenes intercept the excited product of luminol. It is the quenching of chemiluminescence rather than competition with luminol molecules for free radicals, as the magnitude of the constant values shows (Table 2). Such a quenching mechanism and the role of fullerenes in reducing the fluorescence signal are still unclear [54]. In some cases, fullerenes and fullerenols can non-covalently bind molecules, e.g., Ribonuclease A [52] exhibiting static quenching. However, binding sites in each case are individual; for a more detailed study of the binding nature, molecular dynamics simulations should be performed [55]. We evaluated quenching for an ABAP–luminol mixture and luminol alone (see the Supporting information). In both cases, we observed comparable values of Stern–Volmer quenching constants for C_60_, C_70_, and Gd@C_82_ as C_70_ > C_60_ > Gd@C_82_. This quenching can be explained by the average polarizability (α) of fullerene molecules. The polarizabilities as α_EMF_ < α_atom_ + α_Fullerene_ are 114.67 Å^3^ for Gd@C_82_ [56], 102.7 Å^3^ for C_70_ [57], and 82.7 Å^3^ for C_60_. Thus, the higher quenching efficiency of C_70_ and Gd@C_82_ can be attributed to its higher polarizability [58]. In addition, the reactivity upon A_E_-type reactions for fullerenes decreased from C_60_ to Gd@C_82_ [59] as the presence of endoatoms improves the fullerene antiradical capacity [60]. A 1.3-fold decrease in the quenching constant for Gd@C_82_ could also be due to a different behavior in the chemiluminescence reaction, a change in the quenching mechanism to a bimolecular one [53] or another competing process at multiple positions of the fullerene cage [59].

From the data on antioxidant activity of AFDS of C_60_ and C_70_, the following results can be summed up. AFDs of C_60_ and C_70_ show no pro-oxidant activity in the tested model; thus, they can be used for biomedical applications without any hazardous effect. In our opinion, low activity values and mainly quenching properties mean that AFDs of unmodified C_60_ and C_70_ can be used as control values for testing the antioxidant properties of fullerene derivatives. In such a case, any found activity can be attributed to the added functionality and not the fullerene cage.

The most relevant difference in antioxidant activity is that while C_60_ and C_70_ showed a single process that we attribute to quenching, a second process is revealed in Gd@C_82_. This action in Gd@C_82_ is attributed to the interception of ABAP radicals, i.e., actual antioxidant activity. However, the significant CL quenching intrinsic to Gd@C_82_ as to other fullerenes makes it challenging to separate these two signals. This behavior for Gd@C_82_ AFD can be explained by the presence of a carbon cage containing the inner paramagnetic metal ion Gd^3+^ with a spin of 7/2 or Gd@C_82_^3–^ anion acting as a radical located on the outer shell [61]. Gd@C_82_^3–^ can be involved in free-radical addition reactions, which can change the electronic structure of the inner cluster and affect its configuration [62]. The electron affinity of Gd@C_82_ is more significant than those for pristine C_60_ and C_70_ (1.25 and 1.19 times, respectively_2_). Gd@C_82_ acts as a strong electron donor and acceptor [63], which correlates with the relative efficiencies Gd@C_82_(OH)_22_ > C_60_(OH)_22_ to scavenge various free radicals [19]. It is noteworthy that the concentration of Gd@C_82_ was 4 times higher than C_70_ and C_60_ (17.2 and ~4 μM, respectively). Thus, the effect of the second process in Gd@C_82_ kinetics was more prominent. AFDs C_60_ and C_70_ may show the same antioxidant action, but it was less noticeable due to the concentrations.

Thus, for Gd@C_82,_ we can conclude that it shows no pro-oxidant activity like AFDs of C_60_ and C_70_, which is more relevant as Gd@C_82_ is considered for MRI applications [64]. Additionally, Gd@C_82_ shows medium antioxidant activity; thus, it can exhibit cytoprotective properties, which can be the topic of the subsequent study.

## 4. Materials and Methods

### 4.1. Aqueous Fullerene Dispersion Preparation

Preparation and characterization techniques for aqueous fullerene dispersions were recently described elsewhere [30]. In this work, we used long-term stable AFD of the pristine C_60_, C_70_ (NeoTechProduct LLC (St. Petersburg, Russia), 99+% HPLC-grade); the enriched soot containing the Gd@C_2n_ EMFs (total content of Gd atoms up to 4 wt. % has been synthesized by the evaporation of the composite graphite electrodes compounded by gadolinium in the electric arc reactor as we previously described [65]. The sonication time was increased up to 36 h totally, and an ultrasound probe with a large surface was used (*ca*. 7 cm^2^). The main drawback of this process is a time-dependent accumulation of titanium dioxide nanoparticles (TiO_2_ NPs) due to cavitation sonotrode erosion. It is confirmed by ICP-OES analysis for metal impurities in AFDs (Table S1, Supplementary Materials).

A syringe hydrophilic polyvinylidene fluoride (PVDF) filter has been used to remove particles from AFDs. The filter removes large fullerene nanoparticles (more than 1 μm) and finally cleans out titanium nanoparticles (less than ca. 1 ppb) from the ultrasonic probe. ICP-OES showed that AFDs contain silicon, which cannot be removed by syringe filtering. The details on the aqueous fullerene dispersion preparation and material safety data sheet are summarized in the Supplementary Materials.

### 4.2. Techniques and Reagents

The enhanced chemiluminescence protocol for quantification of the antioxidant potential of AFD C_60_, C_70_, and Gd@C_82_ has been used. The chemiluminescent system consisted of a source of free radicals, cationic 2,2′-azobis (2-amidinopropane) dihydrochloride (ABAP; Sigma, St. Louis, MO, USA), and a chemiluminescent probe, 5-amino-2,3-dihydrophthalazine-1,4-dione. Reference antioxidant compound: Trolox^®^ (±)-6-Hydroxy-2,5,7,8-tetra-methylchromane-2-carboxylic acid (Sigma, St. Louis, MO, USA).

A luminol solution of 1 mM (Sigma, USA) and ABAP solution of 50 mM were prepared by dissolving the weighed samples in a phosphate buffer solution 0.1 M KH_2_PO_4_ at pH 7.4 (Sigma, St. Louis, MO, USA). The total volume in a polycarbonate cuvette was 1.00 mL in all experiments. The stock solution of ABAP (2.5 mM) and luminol (2 μM) in the mixture were added to the buffer solution at 37 °C. Reagent addition order: (1) heated phosphate buffer solution, (2) mixture of ABAP and luminol incubated in the dark at room temperature for 20 min. After reaching a steady-state CL signal level, the AFDs, or Trolox^®^ was added (shown as a sharp decrease in readout signal between 10 and 20 min, Figure 1 and Figure 2). The CL signal was recorded until the new stationary level was reached.

The chemiluminescence signal was recorded up to achieving stationary level, and then an aliquot of the antioxidant solution of Trolox or AFDs was added. The registration was performed until the new steady-state level.

### 4.3. Equipment

The measurements were carried out with a Lum-1200 12-channel chemiluminometer (DISoft, Moscow, Russia). The chemiluminometer detects visible light in a range of 300–700 nm. No bandpass filters were used. Signal processing was performed via PowerGraph 3.3 Professional software (DISoft, Moscow, Russia). The relative standard deviation of chemiluminescence intensity did not exceed 0.05. Fluorolog^®^-2 spectrofluorimeter (Horiba Jobin Yvon, Kyoto, Japan) was used. An Agilent 720 ICP-OES spectrometer (Mulgrave, Australia) with an axial view was used for elemental analysis. The statistical processing of the data was performed with STATISTICA v.10.0 software (StatSoft Inc., Tulsa, OK, USA).

Millex-HV Syringe Filter Unit, 0.22 and 0.45 µm, hydrophilic PVDF, 33 mm, non-sterilized were used for AFD filtration during the preparation process (Merck Millipore, Darmstadt, Germany). Sartorius Proline Plus (Göttingen, Germany) mechanical single-channel pipettors of 10 ÷ 100, 100 ÷ 1000 μL were used for the graduation and preparation of solutions calibrated by ISO 8655-2:2002. The ultrasound probe MEF93.T (LLC MELFIZ-ul’trazvuk, Moscow, Russia) working in a continuous mode of exposure to ultrasonic energy at operating frequency 22.00 ± 1.65 kHz has been used for AFD preparation. A SevenCompact^TM^ pH/Ion S220 pH-meter (Mettler-Toledo AG, Greifensee, Switzerland) was used to prepare the phosphate buffer solution. According to IUPAC recommendation [66], calibration was performed using NIST Traceable standard buffer solutions with pH 1.68, 4.01, 6.68, 9.18, and 11.00 (Hanna Instruments, Woonsocket, RI, USA).

### 4.4. Computer Simulation and Data Handling

The computer simulation was carried out with the specially designed computer program Kinetic Analyzer (by Dr. D. Izmailov). For a set of the predetermined reactions and the initial concentrations of the reactants, the rate constants were selected, providing the maximal curve fitting of experimental and calculated plots. As a criterion for the best curve fitting, the minimum sum of squared residuals was calculated using OriginPro 2015 software (OriginLab Corp., Northampton, MA, USA).

We recorded the kinetics of chemiluminescence of antioxidant action to the moment of depletion of the antioxidant, ~100 min. The total areas and areas for the first 20 min of the reaction were calculated using the functional features of PowerGraph software. Chemiluminograms in the molecular model of generation of organic radicals were used to determine the concentration of half-suppression of the chemiluminescent signal for all AFDs ($c_{1/2}$, μM). The concentration of half-suppression of luminescence ($c_{1/2}$, μM) is a concentration that reduces the area the signal *S* of the response by two times and can hypothetically be taken as a quantitative indicator of the inhibitory activity of a given compound.

## 5. Conclusions

Thus, the antioxidant potential requires the estimation of both the thermodynamic and kinetic parts of this parameter. AFDs of C_60_ and C_70_ show no antioxidant activity in the system of organic radical-induced ABAP decay. We prove that it is not free radical capture but quenching. However, Gd@C_82_ has a dual-action mechanism involving a significant antioxidant action. The results provide insights into the possible mechanism of interactions of fullerenes between free-radicals C_60_, C_70_, Gd@C_82,_ which are fundamental to understanding the potential biomedical effects of AFDs. Evaluating the antioxidant activity of fullerenes is helpful in further evaluation of antioxidant properties in a living cell.

We believe that this work helps create reference materials for further study of the antioxidant properties of functional fullerene derivatives. AFDs of C_60_, C_70_ can be proposed as model substances not exhibiting antioxidant properties. Moreover, the absence of a significant free radical interception effect allows the development of sensors to control impurity composition, acting as a free radical interceptor rather than as a quencher for in vitro and in vivo experiments.

## Figures and Tables

**Table 1:** Antioxidant capacity parameters for aqueous fullerene dispersions: area of suppression of the chemiluminescence signal (*S*s_upp_) normalized to 1 µM of AFDs; Trolox equivalents calculated for 1 µM of AFDs; area of suppression of the chemiluminescence signal for the first 20 min (*S*_20_); and half-maximal inhibitory concentration ($c_{1/2}$ ); *n* = 5, *p* = 0.95.

AFD	Concentration Range, μM	TRAP		Suppression Area for the First 20 min, Linear Fit	$c_{1/2},\;\mu M$
		Normalized *S*_supp_ × 10^–^^6^	Trolox Equivalent, µM		
C_60_	1.8 ÷ 18	0.51	0.31	*S*_20_ = (76 ± 6) × c_Ful_, *r* = 0.9955	6.4 ± 0.3
C_70_	2.0 ÷ 20	0.29	0.18	*S*_20_ = (46 ± 2) × c_Ful_, *r* = 0.9956	11.0 ± 0.4
Gd@C_82_	4.0 ÷ 40	0.12	0.072	*S*_20_ = (28 ± 5) × c_Ful_, *r* = 0.9754	22.6 ± 0.8

**Table 2:** Constants for a one-stage mechanism for C_60_ and C_70_, and two-stage for Gd@C_82._ Initial simulation conditions common for all the studied systems: ABAP, 2.5 mM; luminol, 2 μM; radical of ABAP and the excited product area were absent at the starting points.

Initial Concentrations, μM	C_60_	C_70_	Gd@C_82_	Trolox^®^	Reaction
AO	3.5	4.0	17.2	0, 0.1, and 0.2	Quenching reaction (3a)
AO	n/a	n/a	0.172	n/a	Radical interception reaction (3)
Value of Simulated Constant, μM^−1^ min^−1^					
ABAP → R	1.25			1.70	ABAP decomposition (1)
R + Lum → RLum*	2				Formation of an excited product (2)
RLum* → P + hν	1			4	Luminescence (2a)
AO + R^•^ → …	n/a	n/a	30	10,000	Radical interception reaction (3)
AO + RLum* → …	0.20	0.13	0.13	n/a	Quenching reaction (3a)

## Data Availability

Raw data and samples of the aqueous dispersions of fullerenes and endofullerene compounds are available from the authors.

## Supplementary Materials

The following are available online at https://www.mdpi.com/article/10.3390/ijms22115838/s1, Figures S1 and S2, fluorescent spectra of aqueous fullerene dispersions C_60_, C_70_, and Gd@C_82_ act as a quencher at different systems ABAP-luminol, and only luminol. Table S1, the elemental composition of fullerene dispersions by inductively coupled plasma atomic emission spectroscopy (ICP-OES) and pH measurements.
