# Supplementary material for: Antioxidant Potential of Aqueous Dispersions of Fullerenes C60, C70, and Gd@C82
Source: Int J Mol Sci. 2021 May 29;22(11):5838. doi: 10.3390/ijms22115838 (PMC8199091; doi:10.3390/ijms22115838)
Supplement: Supplementary file 1 [file ijms-22-05838-s001.zip › ijms-1222568-supplementary.pdf]

Supplementary Materials
for

Antioxidant Potential of Aqueous Dispersions of Fullerenes C_{60}, C_{70}, and Gd@C_{82}

by

Ivan V. Mikheev [1,*], Madina M. Sozarukova [1,2], Dmitry Yu. Izmailov [3], Ivan E. Kareev [4], Elena V. Proskurnina [5], and Mikhail A. Proskurnin [1]

[1] Chemistry Department Analytical Chemistry Division of Lomonosov Moscow State University, Moscow 119991, Russia; mikheev.ivan@gmail.com

[2] Kurnakov Institute of General and Inorganic Chemistry, Russian Academy of Sciences, Moscow 119991, Russia; s_madinam@bk.ru

[3] Faculty of Fundamental Medicine, Lomonosov Moscow State University, Moscow 119234, Russia; dizm@mail.ru

[4] Institute of Problems of Chemical Physics of the Russian Academy of Sciences, 142432 Chernogolovka, Moscow Region, Russia; kareev@icp.ac.ru

[5] Research Centre for Medical Genetics, Moscow 115522, Russia; proskurnina@gmail.com

* Correspondence: mikheev.ivan@gmail.com; Tel.: +7(495)939-15-68 ext.101

1 Aqueous fullerene dispersion preparation and material safety data sheet 3
2 Quenching fullerene ability in aqueous media 5
2.1 Quenching of ABAP-Luminol system in the presence of fullerene...... 5
2.2 Quenching of Luminol system in the presence of fullerene 5
3 Reference list 8

1 Aqueous fullerene dispersion preparation and material safety data sheet

Chemiluminescent systems can be influenced by the purity of the objects under study. Moreover, the action of the impurity component can be taken as a false positive mechanism. Thus, we carried out a deep purification of the AFDs. We have used the direct ultrasound-assisted preparation technique for AFDs of C_{60}, C_{70}, and Gd@C_{82} preparation [1] due to fewer impurity components and sedimental stability for more than 20 months.

Our experience with a sonication system for carbonaceous nanomaterials (hydrosols of nanodiamond) reveals titanium impurities [2] even for a short time (e.g., several minutes) of ultrasound treatment. Titanium accumulation rate was proportional to the prolonged ultrasound exposure time [3]. Our previous experiments proved that titanium and silicon are oxide species [4]. However, purification of AFD would not be resolved by now. The main idea of AFDs purification is primarily related to reducing the number of inorganic impurities (nanoparticles of titanium and silicon oxides), even considering the losses of the main component. Contamination of AFDs is associated with metal-carbon interactions [5]. It is essential to choose the correct filter system. We used commercially available syringe PVDF membrane filters of 0.22 or 0.45 µm. As for TiO_2 NPs purification, a PVDF membrane can be used due to the high affinity of TiO_2 for water [6]; the presence of hydroxyl moieties from TiO_2 NPs contributed to improving the hydrophilicity, thereby improving the water flux [7]. As shown in Table S1, filtration with a 0.22 or 0.45 µm PVDF filter completely removes TiO_2 NPs fullerene dispersions.

Moreover, a 0.2 µm PTFE filter has low filtration efficiency toward titanium due to redox reactions between TiO_2 NPs and Pu(III)/Pu(IV) at low pHs [3]. However, for practically neutral AFDs Table S1, the efficiency of PVDF filters is high. The presence of impurities can reduce or increase the effectiveness of antioxidants. The ability of TiO_2 NPs to enhance the production of SAR and alter the antioxidant system in human osteoblast cells is shown [8]. Impurities may also adversely affect the reproducibility of results [9]. To evaluate the efficiency of fullerene antioxidant capacity, preparation of pure dispersion is an essential item.

As the AFD concentration after the combination of filters (prefilter 0.45 µm and primary filter 0.22 µm) is higher than after each filter alone (0.45 or 0.22 µm) (Table S1). Indeed, small pores (0.22 µm) clog faster, thereby reducing the filtration efficiency and, as a consequence, lowering the concentration of the target component [10]. Using prefiltration to increase filter life removes several significant limitations associated with losing fullerenes or endofullerenes. Prefiltration is widely used chromatography good laboratory practice [11].

As for SiO_2 NPs purification, they remain in solution (approximately 65%, Table S1) after PVDF filters. SiO_2 NPs pass through the membrane during filtering with polytetrafluoroethylene (PTFE) membranes in a range of 0.1–1.0 µm of pore size [12]. In our opinion, we find a residual silica as typically silica SiO_2 NPs can be formed with less than 220 nm in diameter [13]. Also, intermolecular and intramolecular interactions deal with probable silica surface with an adsorbed fullerene monolayer [14] and sorption of SiO_2 NPs on the surface of fullerene clusters [15]. SiO_2 NPs increased the capacity of the antioxidant enzymes superoxide dismutase (SOD), catalase (CAT), glutathione reductase (GR), and phenylalanine ammonia-lyase (PAL) in plants [16].

Thus, the total content of SiO_2 NPs is less than the total fullerene content ratio in the stock solution by 10 to 170 times, which remains diluted. Therefore, it should not affect the measurements of antioxidant potential.

Table S1. Elemental composition of fullerene dispersions after preparation by direct ultrasound probe sonication (during 5h short-time duty) by inductively coupled plasma atomic emission spectroscopy (ICP-OES). Slurry sampling introduction in ICP-OES spectrometer. The uncertainty is ± 15%.

**Type of AFD or number of compounds**	**Syringe filter type**	$c_{Fullerene}$, **ppm**	**pH***	***c*, ppb**																			
				**Ag**	**Al**	**As**	**B**	**Ba**	**Be**	**Cd**	**Co**	**Cr**	**Cu**	**Fe**	**Li**	**Mn**	**Mo**	**Ni**	**Pb**	**Si**	**Ti**	**V**	**Zn**
C_{60}	Pristine AFD (no filter using)	1210 ± 50	7.35	<5	290	<5	320	17	<0.1	25	<1	<1	80	75	16	45	273	60	<1	2530	595	32	29
	450 nm	260 ± 20	7.44	<5	<10	<5	105	<1	<0.1	<0.5	<1	<1	7	7	2	5	10	<1	<1	1620	<1	<1	6
	220 nm	90 ± 10	7.40	<5	<10	<5	105	<1	<0.1	<0.5	<1	<1	7	7	2	4	10	4	<1	1600	<1	2.4	6
	Combination of 450 and 220 nm filters	280 ± 24	7.45	<5	<10	<5	105	<1	<0.1	<0.5	<1	<1	7	7	2	4	<5	3	<1	1650	2.3	<1	6
C_{70}	450 nm	120 ± 10	7.55	<5	<10	<5	90	<1	<0.1	<0.5	<1	<1	5	5	2	2	<5	<1	<1	1520	<1	<1	<2
Gd@C_{82}	450 nm	20 ± 4	7.74**	<5	<10	<5	12	<1	<0.1	<0.5	<1	<1	2	8	2	2	<5	<1	<1	1450	<1	<1	<2

*, uncertainty was ± 0.10 pH units;
**, pH of pristine AFD Gd@C_{82} was 7.89.

2 Quenching fullerene ability in aqueous media

2.1 Quenching of ABAP-Luminol system in the presence of fullerene

Interactions between the components of free-radical systems (ABAP, luminol) and aqueous fullerene dispersions (AFD) C_{60}, C_{70}, and Gd@C_{82} have been studied by fluorescent spectroscopy. Stern-Volmer approach (K_{SV}) for quenching fluorescence analysis has been applied. Quenching occurs in static (with constant K_S) or dynamic (with constant K_D) interactions mechanism. To more speciation, fluorescence lifetime measurement should be applied. The magnitude of K_S demonstrates that dynamic quenching cannot account for the decrease in intensity [17].

Normalization of fluorescence data at excitation 350 nm in Stern-Volmer coordinates is

$$\frac{I_0}{I} = 1 + K_{SV}[Q]$$

where I_0 is the fluorescence intensity without a quencher; I is the fluorescence intensity with quencher addition; and Q is quencher concentration (M).

All known data are presented for 293K. Quenching of 1-pyrenencarboxilic acid by tris(malonate)-substituted C_{60} were KSV~4.4÷3.4)×104 M^{-1} [18]; for pristine C_{60} and fullerenol $C_{60}(OH)_n$ with RNase A were 8.2×10^4 M^{-1} and 1.7×104 M^{-1} accordingly [19]; bovine (BSA) and human (HSA) serum albumins interaction between Gd@$C_{82}(OH)_{22}$ 2.3 and 2.5×10^4 M^{-1} accordingly by a dynamic mechanism [20].

In this work, we conducted an estimation of quenching fullerene ability for AFD C_{60}, C_{70}, and Gd@C_{82} in the ABAP, luminol system. The magnitude of constant value was C_{60} ~ C_{70} > Gd@C_{82} (3.7 ± 0.1, 3.8 ± 0.1, and 2.9 ± 0.1)×10^4 M^{-1} which have good accordance with [18-20]. The fluorescence spectra are presented below (

Figure S1). The different values of the Stern-Volmer constants for Gd@C_{82} are due to the different nature of the interactions [21]. It can be electrostatic interaction prevails and hydrogen bonds and van der Waals interactions [22] compared to non-endohedral fullerenes.

2.2 Quenching of Luminol system in the presence of fullerene

In addition, we have estimated of quenching fullerene ability for AFD C_{60}, C_{70}, and Gd@C_{82} in only the luminol added system. The linearity of the Stern-Volmer plot indicates that only one quenching mechanism is possible; it could be either dynamic or static for more speciation lifetime measurements is needed. Luminol quenching by AFDs (Figure S2) have the magnitude of constant value in row C_{70} > C_{60} > Gd@C_{82} (7.4 ± 0.1, 5.7 ± 0.3, and 2.9 ± 0.1)×10^4 M^{-1}. The values have good accordance with ABAP behavior, fullerene systems only for Gd@C_{82}.

Figure S1 and S2 showed that AFDs could be quench both luminol and product of luminol triggered by ABAP radicals.

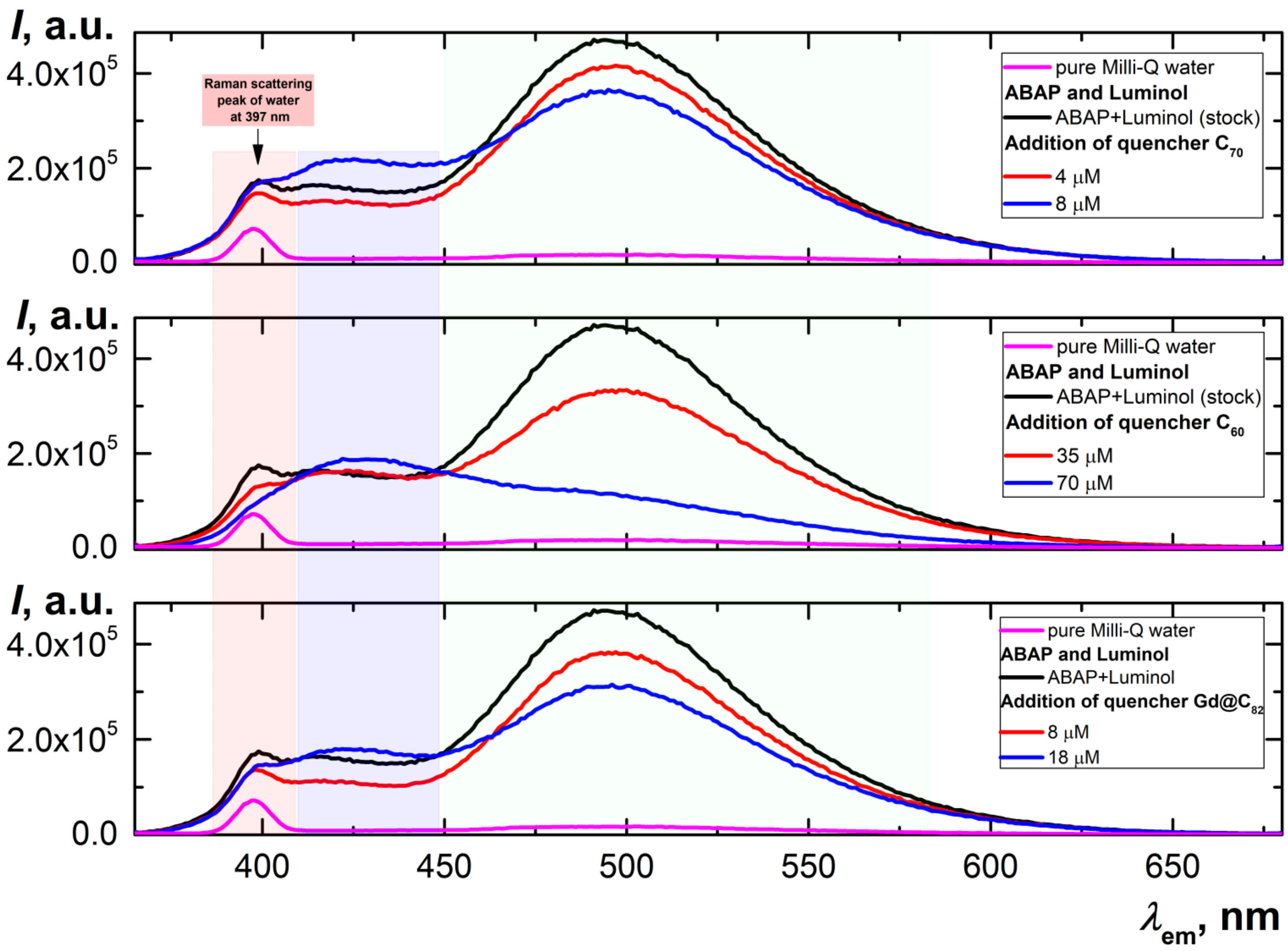

Figure S1. The fluorescent spectra of aqueous fullerene dispersions C_{60}, C_{70}, and Gd@C_{82} act as a quencher at 350 nm excitation wavelength, emission spectra registered 400–700 nm with maxima signal at 494 nm.

Main areas:

(1) Raman scattering peak of water at 397 nm;

(2) transparent blue area is luminol signal ($c_{Luminol}$=2.0 μM);

(3) transparent green area is fluorescence of luminol triggered by ABAP radicals (c_{ABAP}=2.5 mM).

Registration conditions were excitation and emission slits 5 nm, scanning pitch 1 nm, integration time 0.1s, detector voltage 950 V, pH 7.4 (phosphate buffer) at 293K.

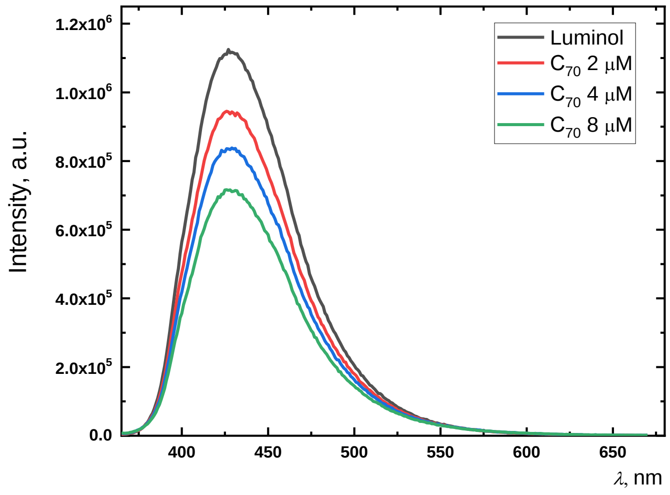

(**a**)

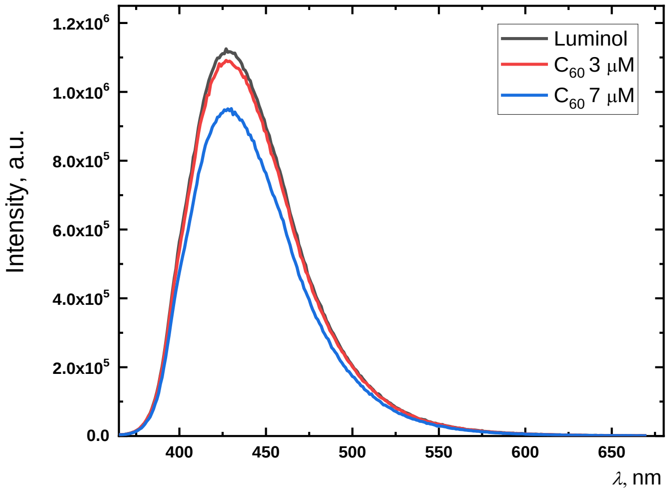

(**b**)

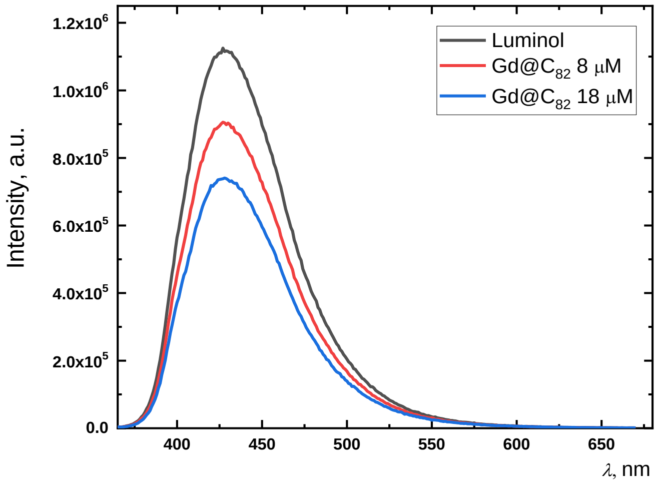

(**c**)

Figure S2.

The fluorescent spectra of luminol ($c_{Luminol}$=2.0 μM) pristine and adding aqueous fullerene dispersions (AFD) C_{60}, C_{70}, and Gd@C_{82} act as a quencher at 350 nm excitation wavelength, emission spectra registered 400–700 nm with maxima signal at 428 nm.

(**a**) AFD C_{60};
(**b**) AFD C_{70};
(**c**) AFD Gd@C_{82}.

Registration conditions were excitation and emission slits 5 nm, scanning pitch 1 nm, integration time 0.1s, detector voltage 950 V, pH 7.4 (phosphate buffer) at 293K.

3 Reference list

1. Mikheev, I.V.; Pirogova, M.O.; Usoltseva, L.O.; Uzhel, A.S.; Bolotnik, T.A.; Kareev, I.E.; Bubnov, V.P.; Lukonina, N.S.; Volkov, D.S.; Goryunkov, A.A.; et al. Green and rapid preparation of long-term stable aqueous dispersions of fullerenes and endohedral fullerenes: The pros and con of an ultrasonic probe. *Ultrason. Sonochem.* **2021**, 105533, doi:https://doi.org/10.1016/j.ultsonch.2021.105533.
2. Myasnikov, I.Y.; Gopin, A.V.; Mikheev, I.V.; Chernysheva, M.G.; Badun, G.A. Presonication of nanodiamond hydrosols in radiolabeling by a tritium thermal activation method. *Mendeleev Communications* **2018**, *28*, 495-496, doi:https://doi.org/10.1016/j.mencom.2018.09.014.
3. Virot, M.; Venault, L.; Moisy, P.; Nikitenko, S.I. Sonochemical redox reactions of Pu(iii) and Pu(iv) in aqueous nitric solutions. *Dalton Transactions* **2015**, *44*, 2567-2574, doi:10.1039/C4DT02330G.
4. V. Mikheev, I.; M. Sozarukova, M.; V. Proskurnina, E.; E. Kareev, I.; A. Proskurnin, M. Non-Functionalized Fullerenes and Endofullerenes in Aqueous Dispersions as Superoxide Scavengers. *Molecules* **2020**, *25*, 2506.
5. Kiciński, W.; Dyjak, S. Transition metal impurities in carbon-based materials: Pitfalls, artifacts and deleterious effects. *Carbon* **2020**, *168*, 748-845, doi:https://doi.org/10.1016/j.carbon.2020.06.004.
6. Martins, P.M.; Miranda, R.; Marques, J.; Tavares, C.J.; Botelho, G.; Lanceros-Mendez, S. Comparative efficiency of TiO2 nanoparticles in suspension vs. immobilization into P(VDF–TrFE) porous membranes. *RSC Advances* **2016**, *6*, 12708-12716, doi:10.1039/C5RA25385C.
7. Samree, K.; Srithai, P.-u.; Kotchaplai, P.; Thuptimdang, P.; Painmanakul, P.; Hunsom, M.; Sairiam, S. Enhancing the Antibacterial Properties of PVDF Membrane by Hydrophilic Surface Modification Using Titanium Dioxide and Silver Nanoparticles. *Membranes* **2020**, *10*, 289.
8. Niska, K.; Pyszka, K.; Tukaj, C.; Wozniak, M.; Radomski, M.W.; Inkielewicz-Stepniak, I. Titanium dioxide nanoparticles enhance production of superoxide anion and alter the antioxidant system in human osteoblast cells. *Int J Nanomedicine* **2015**, *10*, 1095-1107, doi:10.2147/IJN.S73557.
9. Apak, R.; Özyürek, M.; Güçlü, K.; Çapanoğlu, E. Antioxidant Activity/Capacity Measurement. 1. Classification, Physicochemical Principles, Mechanisms, and Electron Transfer (ET)-Based Assays. *Journal of Agricultural and Food Chemistry* **2016**, *64*, 997-1027, doi:10.1021/acs.jafc.5b04739.
10. Japuntich, D.A.; Stenhouse, J.I.T.; Liu, B.Y.H. Effective pore diameter and monodisperse particle clogging of fibrous filters. *Journal of Aerosol Science* **1997**, *28*, 147-158, doi:https://doi.org/10.1016/S0021-8502(96)00064-X.
11. Moldoveanu, S.; David, V. *Modern Sample Preparation for Chromatography*; Elsevier Science: 2021.
12. Ullmann, C.; Babick, F.; Stintz, M. Microfiltration of Submicron-Sized and Nano-Sized Suspensions for Particle Size Determination by Dynamic Light Scattering. *Nanomaterials* **2019**, *9*, 829.
13. Kimoto, S.; Dick, W.D.; Hunt, B.; Szymanski, W.W.; McMurry, P.H.; Roberts, D.L.; Pui, D.Y.H. Characterization of nanosized silica size standards. *Aerosol Science and Technology* **2017**, *51*, 936-945, doi:10.1080/02786826.2017.1335388.
14. Davydov, V.Y.; Sheppard, N.; Osawa, E. An Infrared Spectroscopic Study of the Hydrogenation and Dehydrogenation of the Complexes of Aromatic Compounds and of

Fullerene C60 with Silica-Supported Platinum. *Journal of Catalysis* **2002**, *211*, 42-52, doi:https://doi.org/10.1006/jcat.2002.3694.
15. Chen, K.L.; Elimelech, M. Interaction of Fullerene (C60) Nanoparticles with Humic Acid and Alginate Coated Silica Surfaces: Measurements, Mechanisms, and Environmental Implications. *Environmental Science & Technology* **2008**, *42*, 7607-7614, doi:10.1021/es8012062.
16. Emamverdian, A.; Ding, Y.; Mokhberdoran, F.; Xie, Y.; Zheng, X.; Wang, Y. Silicon dioxide nanoparticles improve plant growth by enhancing antioxidant enzyme capacity in bamboo (Pleioblastus pygmaeus) under lead toxicity. *Trees* **2020**, *34*, 469-481, doi:10.1007/s00468-019-01929-z.
17. Lakowicz, J.R. *Principles of Fluorescence Spectroscopy*; Springer US: 2007.
18. Wang, I.C.; Tai, L.A.; Lee, D.D.; Kanakamma, P.P.; Shen, C.K.F.; Luh, T.-Y.; Cheng, C.H.; Hwang, K.C. C_{60} and Water-Soluble Fullerene Derivatives as Antioxidants Against Radical-Initiated Lipid Peroxidation. *J. Med. Chem.* **1999**, *42*, 4614-4620, doi:10.1021/jm990144s.
19. Roy, P.; Bag, S.; Chakraborty, D.; Dasgupta, S. Exploring the Inhibitory and Antioxidant Effects of Fullerene and Fullerenol on Ribonuclease A. *ACS Omega* **2018**, *3*, 12270-12283, doi:10.1021/acsomega.8b01584.
20. Liu, X.; Ying, X.; Li, Y.; Yang, H.; Hao, W.; Yu, M. Identification differential behavior of $Gd@C_{82}(OH)_{22}$ upon interaction with serum albumin using spectroscopic analysis. *Spectrochimica Acta Part A: Molecular and Biomolecular Spectroscopy* **2018**, *203*, 383-396, doi:https://doi.org/10.1016/j.saa.2018.05.125.
21. Sharoyko, V.V.; Ageev, S.V.; Podolsky, N.E.; Petrov, A.V.; Litasova, E.V.; Vlasov, T.D.; Vasina, L.V.; Murin, I.V.; Piotrovskiy, L.B.; Semenov, K.N. Biologically active water-soluble fullerene adducts: Das Glasperlenspiel (by H. Hesse)? *Journal of Molecular Liquids* **2021**, *323*, 114990, doi:https://doi.org/10.1016/j.molliq.2020.114990.
22. Yang, L.-Y.; Hua, S.-Y.; Zhou, Z.-Q.; Wang, G.-C.; Jiang, F.-L.; Liu, Y. Characterization of fullerenol-protein interactions and an extended investigation on cytotoxicity. *Colloids and Surfaces B: Biointerfaces* **2017**, *157*, 261-267, doi:https://doi.org/10.1016/j.colsurfb.2017.05.065.
